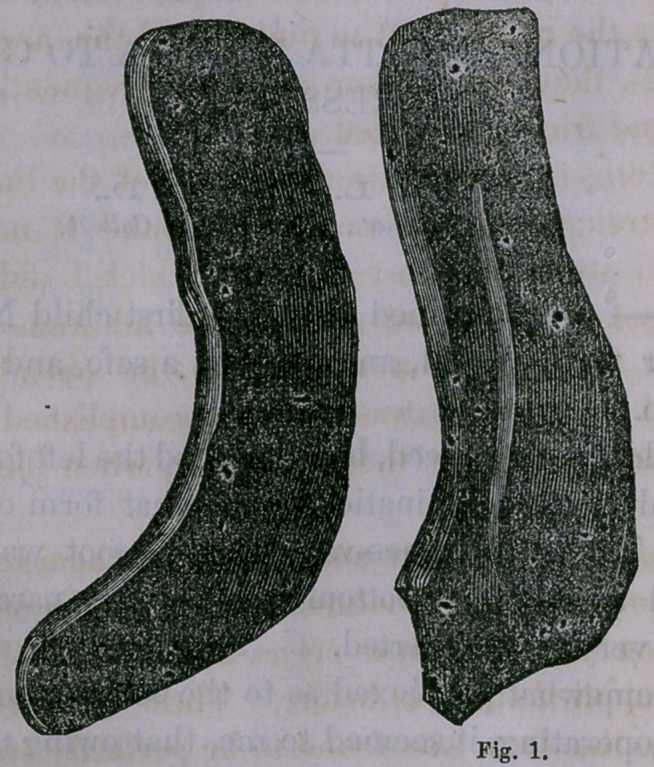# Application of Gutta Percha to Club Foot Dressings

**Published:** 1861-04

**Authors:** R. L. Rea

**Affiliations:** Prof. of Anat. Rush Med. College


### MEDICAL JOURNAL.

VOL. IV.] APRIL, 1861. [NO. 4.

### ORIGINAL COMMUNICATIONS.

### APPLICATION OF GUTTA PERCHA TO CLUB FOOT DRESSINGS.

BY R. L. REA, M. D.,

*Prof. of Anat., Rush Med. College.*

Mrs. —— was confined with her first child Nov. 5, 1860. The labor was natural, and she had a safe and satisfactory getting up.

The child I soon noticed, however, had the left foot deformed, and found upon examination it had that form of Club foot known as Talipes calcaneo-valgus. The foot was drawn outward and upward, the bottom being nearly parallel with the limb and very much everted.

I was somewhat perplexed as to the best method to pursue; for, after operating, it seemed to me, that owing to the tenderness of the skin, it would be next to impossible to retain any of the ordinary dressings with sufficient constancy to warrant the expectation of much from them. The probability that the difficulty would rather increase than diminish by time, and the impressible parts be assuming permanent forms, decided me to adopt some temporary dressing in order to prevent, as far as possible, an increase of the deformity.

Gutta Percha suggested itself, from its perfect flexibility and adaptiveness when softened by heat, and the more I reflected on it, the more I became convinced that that dressing would answer most admirably the intended purpose, and perhaps supersede the necessity of an operation altogether.

Accordingly, after waiting until the mother who had not known it, had so far recovered as to prevent any unfavorable effect from the cheerless news, I proceeded, Nov. 21, to dress the foot in the following manner:

I procured some Gutta Percha of the ordinary sheet kind as kept in the shops, one-eighth of an inch thick. Then, first cutting a pattern of paper, I formed two splints, as represented in the adjoining cuts:

Fig. 1.

They will be seen to consist of two half gaiters, which extend as splints up the leg almost to the knee, the gaiter portions being large enough to almost completely embrace the foot when adjusted.

The splints were then softened in hot water until perfectly flexible and yielding. The limb being well oiled, they were applied and accurately moulded to the parts; the foot was then bent to occupy the desired position, and while held firmly thus, was dipped in cold water, which, rapidly cooling the splints, fixed the limb securely in position indicated.

I then removed the splints, and, after drying, lined them with several thicknesses of canton flannel, when I re-applied them, fastening them carefully with the ordinary roller bandage.

This dressing was examined once in two or three days, to see that it was not chafing, and carefully re-adjusted, the limb at each examination being well bathed in cold water, followed by vigorous friction.

A week or ten days was sufficient to prove to me the wisdom of my course, a very manifest improvement being perceptible. As the mother got to understand the application of the dressings, they were taken off more frequently, and the cold water and frictions applied.

On Dec. 26th, from the natural warmth of the limb and the increasing strength of the muscles, I found it necessary to remodel and strengthen my dressing, which I did as represented in fig. 1 of the wood cuts, by the addition of another thickness of Gutta Percha applied to the outside of those splints I had used, which was readily accomplished by warming the two and applying them together, when they adhered firmly.

After softening the splints and adjusting them to the limb, I bent the foot beyond the natural straight shape of the limb, approximating that of Talipes varus, and after cooling, fixed, padded and applied them as before. These were worn for six weeks, when the foot was found to be perfectly straight and natural in every particular, so much so that the most careful examination could not distinguish the affected foot, when the dressing was discontinued. It is now two months since the dressing was used, and there is not the slightest tendency to return.

It might be proper to mention that the characteristic attendant of flattening of the sole of the foot, from sinking of the plantar arch, was present in a very marked degree, and was removed with the rest of the deformity.

These splints never gave rise to the slightest abrasion or irritation, taking as they did the exact mould and shape of the limb, giving equable pressure at all points. In addition it has another most desirable quality, that of cleanliness, not absorbing moisture, and can be cleansed without difficulty.

This dressing for cases to which it is applicable, it occurs to me, has this very obvious merit: it allows the surgeon to apply effective restraint to the distorting muscles, much earlier than he could otherwise do, owing to the tenderness of the infant skin. This early application of restraint gives the surgeon the paramount advantage in time, before the yielding and less resisting parts, become fixed in their abnormal shape, for it has been abundantly shown that textural alterations almost, if not always, occur subsequent to birth, and the earlier the remedial applications, the more confidently may satisfactory results be expected

This dressing, it seems to me, would be quite as applicable to cases where the deformity was so great, or permanent, as to render resort to the knife necessary. They could be modified in shape so as to embrace any required part of the foot, or strengthened by an increase of thickness, either by adding layers externally or having it manufactured of the right strength, so as to adapt them to any required case. Or, they could be strengthened by the addition of conforming splints of steel or wood externally, which, while they would still retain their most desirable feature of uniform pressure, would give the additional strength of these substances.

*Surgical Anatomy.*—Writers describe four forms of club foot, according to the muscles acting to cause the defect, and the usual monotony in the study of the muscles is somewhat broken up in the group of the leg and foot, inasmuch as they are the active agents in producing this unsightly deformity. The most simple of these forms is Talipes equinus, in which the heel is raised from the ground by the rigid contraction of the Gastrocnemius and Soleus muscles, the weight of the body being thrown forward on the ball of the foot. It is remedied by dividing the tendo Achillis, (the tendon of those two muscles,) an inch above its insertion into the Os calcis.

Probably the most rare variety is the Talipes calcaneus, in which, by a contraction of the extensors, the toes are raised and the weight of the body is thrown on the heel. The Extensor, Prop. Policis, Et. Longus Digitorum, Peroneus Tertius and Tibialis Anticus, are the muscles acting wholly or in part to produce this deformity. They may be divided after they pass beneath the annular ligament, recollecting the situation of the Dorsalis Pedis artery and Anterior Tibial nerve, between the tendon of the Extensor Proprius Policis and the innermost tendon of the Extensor Long. Digitorum; the nerve lying to the outside of the artery.

Talipes valgus, the deformity present in the case I have described, consists in the turning of the foot inward by the action of the Peroneus Longus and Brevis muscles, so that the weight of the body rests on the inner side of the foot and ankle, and is seldom a simple form, being most frequently combined with a greater or less degree of T. calcaneus, forming what might be called T. calcaneo-valgus. The sole of the foot is usually flattened, from flaccidity of the plantar ligaments allowing the plantar arch to give away. This form is remedied by division of these tendons, (Peronei), which can be most readily and safely done below and in front of the outer malleolus upon the outer side of the Os calcis, where they both lie together and can be divided with little difficulty or danger.

By far the most common variety is the Talipes varus, in which the foot is inverted, so that the weight rests on the outer side of the foot and ankle, and, as we find in T. valgus, is seldom a simple form, being most frequently combined with T. equinus, the foot thrown forward, the weight coming on the anterior outer side of the foot.

The Tibialis Anticus, passing as it does in front of the inner malleolus, to be inserted into the adjacent portions of internal cuneiform, and metatarsal bone of the great toe, on their inner sides, by its action will elevate the inner border of the foot, while the Tibialis Posticus passing behind the same malleolus to its insertion in the inner extremity of the Scaphoid, will twist the foot inwards and upon itself, and acting with the Flexor Longus Digitorum, throw the foot forward, causing it to rest, as it does most commonly, on the anterior portion of its outer side.

The plantar fascia is in most cases contracted, and will need to be divided across. Section of the tendon of Tibialis Anticus may be made just previous to its insertion, though the tendon may be distinguished more readily nearer the annular ligament, as it flattens out very much just before it is inserted. Tibialis Posticus may be divided a half inch below and in front of the point of the inner malleolus, being careful not to allow the point of the knife to wound the Internal Plantar artery, as it passes along the inner margin of the sole of the foot. Or this tendon, as well as that of the Flexor Long. Digitorum, may be divided, as they pass close behind the inner malleolus, lying together, and in front of the other important tissues.

There will be very many combinations of the actions of these, and other muscles, producing the different deformities found in Club Foot—to distinguish which, must be left to the intelligence and discrimination of the Surgeon.

## Figures and Tables

**Fig. 1. f1:**